# Variable intraspecific space use supports optimality in an apex predator

**DOI:** 10.1038/s41598-021-00667-y

**Published:** 2021-10-26

**Authors:** S. P. Finnegan, N. J. Svoboda, N. L. Fowler, S. L. Schooler, J. L. Belant

**Affiliations:** 1 Global Wildlife Conservation Center, State University of New York College of Environmental Science and Forestry, 1 Forestry Drive, Syracuse, NY 13204 USA; 2 Alaska Department of Fish and Game, Kodiak, AK USA; 3 Alaska Department of Fish and Game, Soldotna, AK USA

**Keywords:** Ecology, Zoology

## Abstract

Within optimality theory, an animal’s home range can be considered a fitness-driven attempt to obtain resources for survival and reproduction while minimizing costs. We assessed whether brown bears (*Ursus arctos*) in two island populations maximized resource patches within home ranges (Resource Dispersion Hypothesis [RDH]) or occupied only areas necessary to meet their biological requirements (Temporal Resource Variability Hypothesis [TRVH]) at annual and seasonal scales. We further examined how intrinsic factors (age, reproductive status) affected optimal choices. We found dynamic patterns of space use between populations, with support for RDH and TRVH at both scales. The RDH was likely supported seasonally as a result of bears maximizing space use to obtain a mix of nutritional resources for weight gain. Annually, support for RDH likely reflected changing abundances and distributions of foods within different timber stand classes. TRVH was supported at both scales, with bears minimizing space use when food resources were temporally concentrated. Range sizes and optimal strategies varied among sex and reproductive classes, with males occupying larger ranges, supporting mate seeking behavior and increased metabolic demands of larger body sizes. This work emphasizes the importance of scale when examining animal movement ecology, as optimal behavioral decisions are scale dependent.

## Introduction

Animal space use is influenced by numerous factors including presence of competitors, effects of predation, spatial arrangement of resources, and their temporal availability^1,2^. Animal movement leading to home range selection can directly influence survival and fitness through energy gain, resource acquisition, predation avoidance and reproduction, and thus is strongly influenced by natural selection^3^. According to ideal free distribution theory, animals move freely among habitats, positioning themselves and establishing ranges in proportion to resource availability^4^. The establishment of a home range can be considered a fitness-motivated attempt to obtain the resources necessary for survival and reproduction, while minimizing costs^5^. This behavior falls under the theory of optimality, wherein animals maximize resources within their range (resource maximization) or use the minimum area necessary to meet their energetic requirements (area minimization)^6^.

Temporal availability and spatial distribution of resources can alter animal space use under resource maximizing and area minimizing strategies^5^, and animals may restrict their movements when resources are spatially or temporally abundant^7^. An animal using a resource maximization strategy will obtain as many resources as feasible against the associated cost of including more resource rich patches within their home range^8^. In contrast, area minimizers occupy only the amount of space necessary to gain resources essential for reproduction and survival^1^. Including more resource rich patches within a home range will eventually result in a threshold whereby additional patches would not increase individual fitness^8^. Additionally, animals may shift between optimal strategies based on intrinsic (e.g. reproductive status, sex, and age) and extrinsic (e.g. resource dispersion, landscape heterogeneity) factors^1^.

Food availability is arguably the most important factor that influences the size and location of home ranges within a population^9^. The resource dispersion hypothesis (RDH) indicates that an increase in resource patchiness may result in animals requiring larger areas to meet their energetic requirements^10–13^. Although high levels of resource dispersion often occur naturally in heterogeneous landscapes, habitat fragmentation from anthropogenic sources has resulted in increased variability of resource distribution in many ecosystems (e.g.^8^). Mitchell and Powell^1^ suggested that intermediate levels of fragmentation had the greatest influence on animal home range size, resulting in larger ranges for animals using an area minimizing strategy. Additionally, seasonal variation in resource availability can alter animal space use^14^. The temporal resource variability hypothesis (TRVH) states that animal movements and home range sizes decrease during seasons of increased nutritional availability^15^. Under both hypotheses, animals may gain advantages by employing an area minimizing strategy when resources are concentrated, or a resource maximization strategy when resources are more widely distributed^1^.

A positive relationship exists between mammalian body size and home range size^16–18^, with larger species often requiring larger areas to obtain adequate resources to meet increased energetic demands^19^. Among species that display sexual size dimorphism, such as brown bears (*Ursus arctos*), larger males often occupy larger home ranges than smaller females^19,20^. Although likely due to greater energetic demands, it may also reflect mate-seeking behavior, whereby males use larger ranges to increase reproductive success^21^. Larger-bodied individuals often have greater access to resources, such as terrestrial meat^14^. The distribution of meat-based resources, such as ungulates, may result in decreased bear range sizes, particularly during seasons of increased availability (calving season, hunting season) as bears depredate vulnerable neonates or scavenge on carcasses^22,23^.

Differences in space use between male and female brown bears may also be explained by the infanticide avoidance hypotheses^21^. Infanticide is the killing of conspecific young to increase an individual’s reproductive opportunities and is a commonly reported occurrence among brown bears^24,25^. Consequently, female bears with dependent young may restrict movements and home range size to reduce risky encounters with males and solitary females^21^. Alternatively, females with young may increase home range size to maximize available resources to meet their increased energetic demands^19^. Age can influence home range size in mammalian species due to age-related individual dominance among older individuals^20^. Age is closely associated with body size, where older individuals commonly have greater body sizes until reaching an asymptote^26^, often being more dominant and thus holding larger territories with greater access to resources^20^.

**Figure 1:**
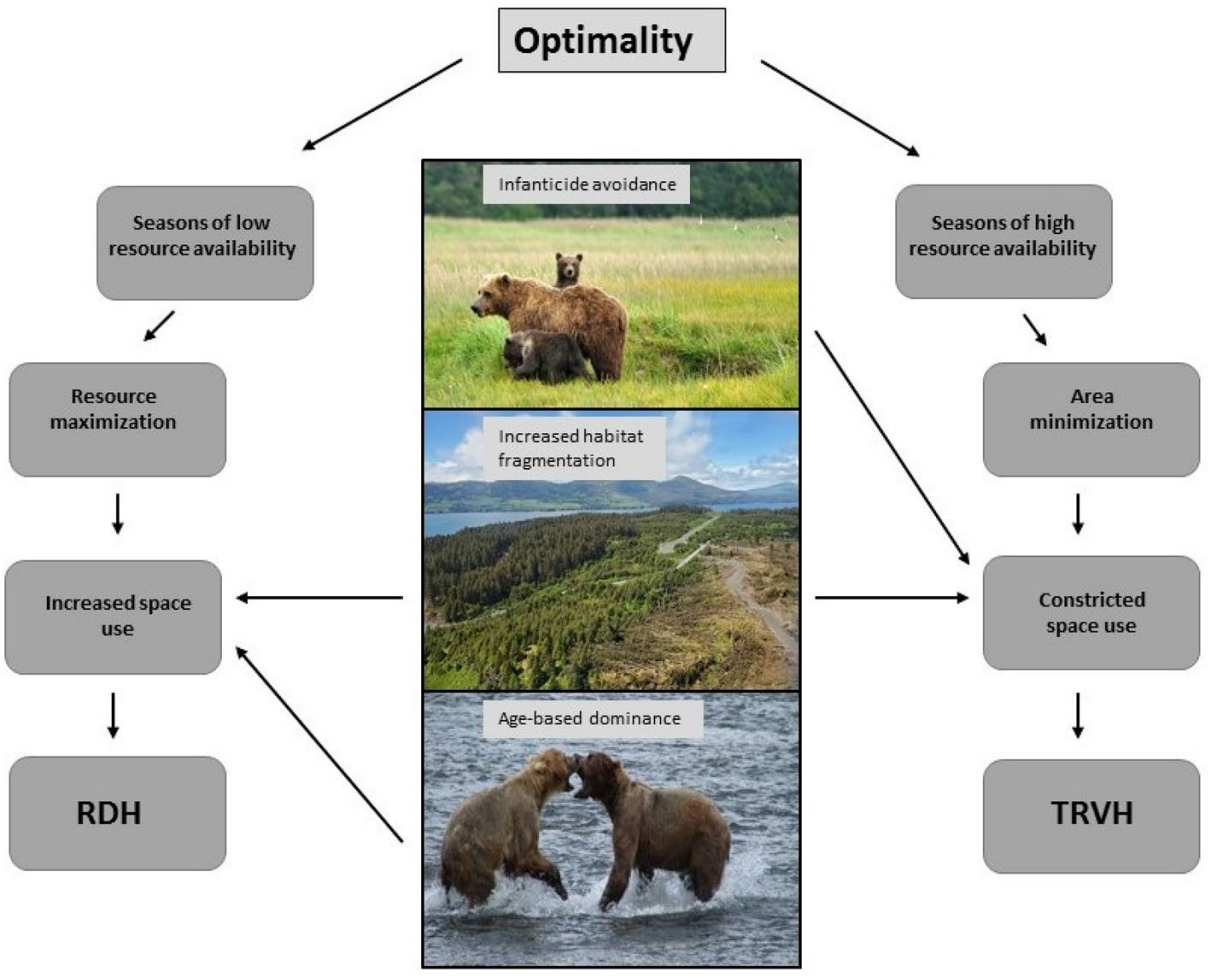
Conceptual model of potential brown bear seasonal movements within an optimality framework. Photo credits Kodiak Brown Bear Trust.

Brown bears are dietary generalists and occupy diverse ecosystems^27–29^ exhibiting considerable temporal variation in diet that can influence space use^30^. Under the optimality paradigm, we assessed whether bear home range size was correlated with (RDH) and/or (TRVH) (Fig. 1). We predicted that optimality would be scale dependent, with divergent patterns between annual and seasonal scales based on resource availability. Furthermore, we expected brown bears to follow the RDH after den emergence (spring) and before hibernation (fall) when resources are more limited and dispersed. We also predicted under the TRVH that bears would use an area minimization strategy during periods of high resource availability (summer). We predicted different strategies between males and females, with females occupying smaller and more productive ranges to meet increased nutritional demands of reproduction. To reduce the risk of infanticide, we expected females with dependent young to hold smaller ranges compared to males and solitary females. Lastly, we predicted older individuals with larger body sizes would occupy larger ranges as they can dominate resource rich areas over younger individuals. Alternatively, older individuals may also occupy smaller ranges with access to areas containing the most abundant resources.

## Materials and methods

### Study area

**Figure 2:**
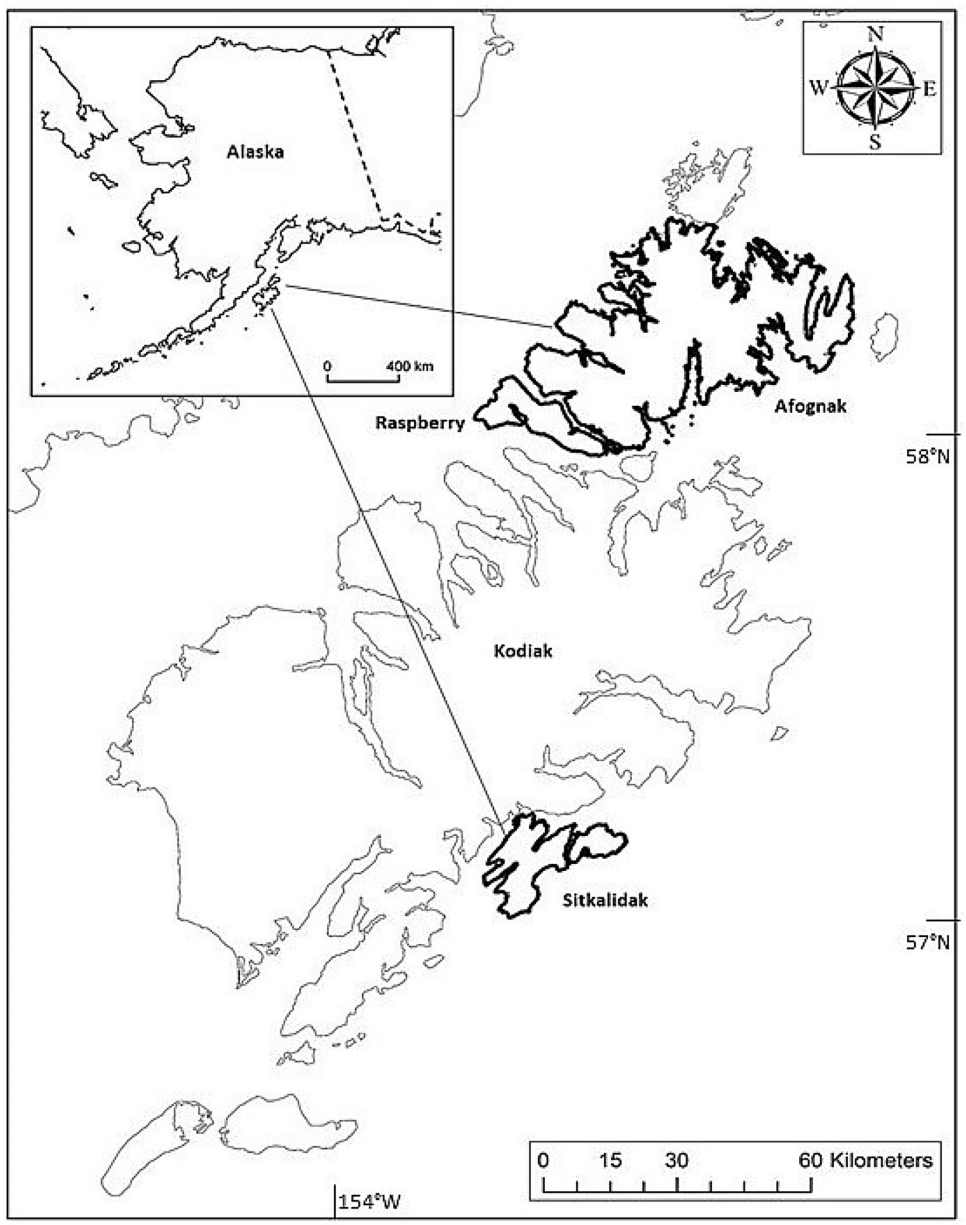
Location of Afognak, Raspberry and Sitkalidak islands, Kodiak Archipelago, Alaska, USA. Map generated with ArcGIS 10.5, ESRI 2020, https://www.arcgis.com/index.html.

Afognak (58.3279° N, 152.6415° W) (1809 km^2^) and Raspberry (58.0708° N, 153.1876° W) (197 km^2^) islands are in the Kodiak Archipelago, Alaska, USA, 5 km north of Kodiak Island and separated by a 1.5-km wide strait (Fig. 2). Both islands contain rolling mountains, with elevations to 739 m on Afognak and 732 m on Raspberry. Average annual rainfall and snowfall for the archipelago are 198 cm and 189 cm, respectively^31^. The archipelago has a subarctic maritime climate with average annual high and low temperatures of 7.9 °C and 1.9 °C, respectively^31^. Sitka spruce (*Picea stichensis*) is the dominant tree species on Afognak, while devil’s club (*Oplopanax horridus*), blueberry (*Vaccinium ovalifolium*), salmonberry (*Rubus spectabilis*), and willow (*Salix* spp.) are dominant understory species. Chum (*O. keta*), coho (*O. kisutch*), pink (*O.gorbuscha*), and sockeye (*O. nerka*) salmon migrate and spawn throughout the island’s streams and lakes. Afognak Island was commercially logged during the 1930s and since 1979, however, no commercial logging has occurred on Raspberry Island since the 1930s. Logging on Afognak occurred during this study. Both islands are owned by native corporations (64%) and state (27%) and federal (9%) governments. Roosevelt elk (*Cervus canadensis roosevelti*) occur on both islands since introduction to Afognak in 1929, and first observations on Raspberry in 1951^32^. Sitka black-tailed deer (*Odocoileus hemionus sitkensis)* occur throughout the archipelago since their introduction in the 1890s, and are the primary terrestrial meat source available to brown bears^33^. Because of close proximity and presumed inter-island movements of brown bears and elk, we considered Afognak and Raspberry islands a single study site, hereafter referred to as Afognak.

Sitkalidak Island (57.1030° N, 153.2356° W, Fig. 2) (300 km^2^) is separated from Kodiak Island by a 320–3200-m wide strait and lies about 91 km South of Afognak Island. Sitkalidak Island has deep fjords and steep mountains, with elevations peaking at 672 m, and is covered with grasses, alder (*Alnus* spp*.)* and willow (*Salix* spp.). Several streams provide spawning habitat for five species of Pacific salmon. Sitkalidak Island is owned primarily by Old Harbor Native Corporation and does not contain elk, however a small herd of bison (*Bison bison*) were introduced in 2017. Presently, no commercial logging or large scale anthropogenic activities occur on Sitkalidak Island. On Afognak and Sitkalidak islands, brown bears rely seasonally on salmon, vegetation and berries^33–35^, and, to a lesser degree, ungulate prey and other marine derived nutrients^34,36^.

### Animal handling

We captured bears and elk during 2017–2020 using standard aerial darting techniques with an R44 helicopter and rifle-fired (CapChur SS cartridge-fired rifle) darts containing Telazol (Zoetis Services LLC; Parsippany, USA) for bears^37^. For elk immobilization we used etorphine (ZooPharm; Laramie, USA) and xylazine (Bimeda Animal Health, Dublin, Ireland)^38^ in 2017, carfentanil (ZooPharm; Laramie, USA) and xylazine (Bimeda Animal Health, Dublin, Ireland)^39^ in 2018 and thiafentanil oxalate (Wildlife Pharmaceuticals Pty Ltd, White River, South Africa) and xylazine (Bimeda Animal Health, Dublin, Ireland) in 2019/ 2020^40^. We fitted animals with global positioning system (GPS) collars (model TGW-4677; Telonics, Inc., Mesa, Arizona, USA and model Vertex Plus-4, Vectronic, Berlin, Germany). We programmed collars to attempt a relocation every 60 min and used a collar release programmed to release collars 21–24 months post capture and inserted a leather link designed to degrade after 2 years^41^. We extracted a vestigial upper premolar from bears to estimate age using cementum annuli counts^42^. For both species we recorded sex and for females, evidence of lactation and presence of young. We administered a combination of naltrexone (Wildlife Pharmaceuticals Pty Ltd, White River, South Africa, or ZooPharm; Laramie, USA) and atipamezole (ZooPharm; Laramie, USA) to reverse elk following handling. We positioned bears sternal following handling to recover at their capture sites. All captures were carried out by experienced team members with approval from a state veterinarian. All animal handling procedures were approved by and followed State University of New York College of Environmental Science and Forestry Institutional Animal Care and Use (IACUC) (protocol 180503), Mississippi State University Institutional Animal Care and Use (IACUC) (protocol 17-236) and Alaska Department of Fish and Game (ADFG; IACUC protocol 0030-2017-37). The study was carried out in compliance with the ARRIVE guidelines. All methods were carried out in accordance with relevant guidelines and regulations.

### Data analysis

To determine brown bear space use we collated data by year for individuals into three seasons: spring (den emergence, April–June), summer (duration of salmon availability, July–September) and fall (pre-denning, October–November). We excluded data from individuals 5 days post capture to account for potential recovery effects on movement behavior^43^, and excluded the day collars were programmed to drop off. We also omitted data after bears entered dens for hibernation. We included only individuals with > 60 locations in a given season^14^ and > 300 locations annually. We estimated 50% utilization distributions (UD) using an autocorrelated kernel density estimator (AKDE^44^). We clipped UDs along coastlines to exclude areas of ocean^45,46^. This resulted in an average reduction of Afognak UD’s by 11% and Sitkalidak UD’s by 23%.

### Habitat variables

To describe variation in resource use we used land cover/vegetation classification produced by digital image analysis of a 3-date temporal composite of Landsat ETM+ scenes^47^. This data set includes national elevation data (NED) and national hydrology data (NHD). This 30-m resolution land cover/vegetation data includes 64 distinct cover types, which we aggregated into six classes (forest, shrub, dwarf-shrub, meadow, wetland, and non-vegetated). We obtained timber harvest data on Afognak Island from Afognak Native Corporation, Koniag Native Corporation, Koncor, Natives of Kodiak Native Corporation, and Ouzinkie Native Corporation. We characterized forest stands by age including clear cut (0–5 years), open regeneration (6–20 years), young forest (21–40 years), mature forest (41–60 years), and old forest (> 60 years)^48^. To assess spatial configuration of resources and evaluate fragmentation we calculated edge density (eight direction sum of all edges of a particular habitat class in relation to landscape area), total patch area (eight direction sum of the area of all patches in the landscape) and mean of patch area (eight direction sum of each class as the mean of all patch areas belonging to a given class) for each UD using the package ‘landscapemetrics’^49^ in program R. We combined four classes of shrub and dwarf shrub habitat as an indication of berry habitat, and calculated the percentage of this berry habitat in each UD. We included berry habitat, as fruit producing species are an important food of brown bears on the archipelago^36^. We calculated the proportional percentage of each forest stand class occurring within a UD. We calculated Shannon’s diversity index as a relative measure of cover diversity (e.g., patch richness, patch evenness) within each UD^50^. Due to the importance of coastal shorelines and salmon spawning streams for foraging^51^, we also included the distance from the center of each UD to nearest coast and salmon stream as covariates in resource modeling.

### Elk RSF

As neonate ungulates can be an important food for brown bears^52^, and bears may scavenge elk carcass remains during hunting seasons, we examined whether UD size was affected by seasonal occurrence of female elk using a resource selection function (RSF)^53^. We determined annual space use for female-collared elk by collating GPS points for individuals with data during April–November for use in brown bear annual models. We calculated elk seasonal space use during the calving (15 May–30 June), summer (1 July–20 September), and hunting (21 September–10 November) periods. These periods reflect times of potential ecological importance to elk, and thus differ from bear seasons. We used the same measures for data exclusion as described for bears and determined annual and seasonal UDs using 95% AKDE. This method uses a generalization of the conventional Gaussian reference function, and accounts for serial autocorrelation within the data, thus eliminating the need to subsample the data set. We implemented AKDE using the R package “ctmm”^54^. We clipped UD’s along coastlines and within each annual UD. For each individual we created random points at a 5:1 available (absence) to used (presence) ratio^55^. For seasonal analysis, we used the boundaries of the annual UD and created available points based on the number of used seasonal locations at a 5:1 ratio. To describe habitat selection of elk we attributed each location to one of six habitat classes (forest, shrub, dwarf-shrub, meadow, wetland, and non-vegetated) and recorded elevation for each animal location. We used a generalized linear mixed effects model for each season and year with animal ID as a random effect, habitat variables and elevation as model covariates and presence/absence as the response. To test model power we calculated an area under the curve (AUC) score for all models and excluded models with an AUC score of < 0.6^56^. We created female elk probability of use maps with 30-m resolution, with each cell containing a value between 1 and 0 (1 = highest probability of use) and incorporated these as a covariate within respective bear models.

### Models

We used a Pearson’s product-moment correlation coefficient (r) to test for multicollinearity among independent variables. We assumed that multicollinearity did not influence model results if |r| < 0.70 for any pair of independent variables^57^. If multicollinearity occurred, we did not include both variables in the same model. We assessed whether data were normally distributed, and after examination of our response variable (UD area), we detected non-normality and performed a log transformation. We created a set of a priori models based on predictions of ecological relevance for each study site including 10 candidate models to test the RDH and TRVH for each season and annually on Afognak (40 total), and seven candidate models for Sitkalidak (28 total) (Table 1). We ran 68 linear models to examine annual and seasonal differences in bear UDs. Top models were selected based on lowest Akaike Information Criterion adjusted for small samples sizes (AICc) for all model combinations^58^. We included null models for comparison, and only models with Δ_*i*_ ≤ 2 were selected for further consideration. We used R v.4.0.2^59^ for statistical analyses.

**Table 1:** Set of a priori models to test the resource dispersion hypotheses (RDH) and the temporal resource variability hypotheses (TRVH) on brown bear annual and seasonal range size, Afognak and Sitkalidak islands, Alaska, USA, June–November 2017–2020.

Island	Model	Variables
Afognak	TRVH1	Distance to salmon stream, distance to coast, elk RSF, age, reproductive status, year
	TRVH2	Berry habitat, age, reproductive status, year
	TRVH3	Elk RSF, age, reproductive status, year
	TRVH4	Distance to salmon stream, distance to coast, age, reproductive status, year
	RDH5	Shannon’s diversity, age, reproductive status, year
	RDH6	Timber stand ages, berry habitat, mean of patch area, age, reproductive status, year
	RDH7	Timber stand ages, berry habitat, edge density, age, reproductive status, year
	RDH8	Timber stand ages (excluding 60+ years), berry habitat, total patch area, edge density, age, reproductive status, year
	Sex and age	Age, reproductive status
	Null	
Sitkalidak	TRVH1	Distance to salmon stream, distance to coast, age, reproductive status, year
	TRVH2	Berry habitat, age, reproductive status, year
	RDH5	Shannon’s diversity, age, reproductive status, year
	RDH6	Berry habitat, total patch area, mean of patch area, age, reproductive status, year
	RDH7	Berry habitat, edge density, mean of patch area, age, reproductive status, year
	Sex and age	Age, reproductive status
	Null	

Model parameter definitions are; Timber stand ages = 0–5 years, 6–20 years, 21–40 years, 41–60 years, and > 60 years for Afognak Island; *Berry habitat* % berry cover within each UD, *Edge density* sum of all edges of a particular habitat class in relation to landscape area for each UD, *Total patch area* the sum of the area of all patches in the landscape within each UD, *Shannon’s diversity* Shannon’s diversity index within each UD, *Mean of patch area* summary of each class as the mean of all patch areas belonging to a given class within each UD, *Salmon stream* average distance to salmon streams from UDs, *Coast* average distance to coast from UDs, Elk Resource Selection Function (*RSF* average probability of elk occurrence within each UD, *Reproductive status* males, females, females with young).

## Results

**Figure 3:**
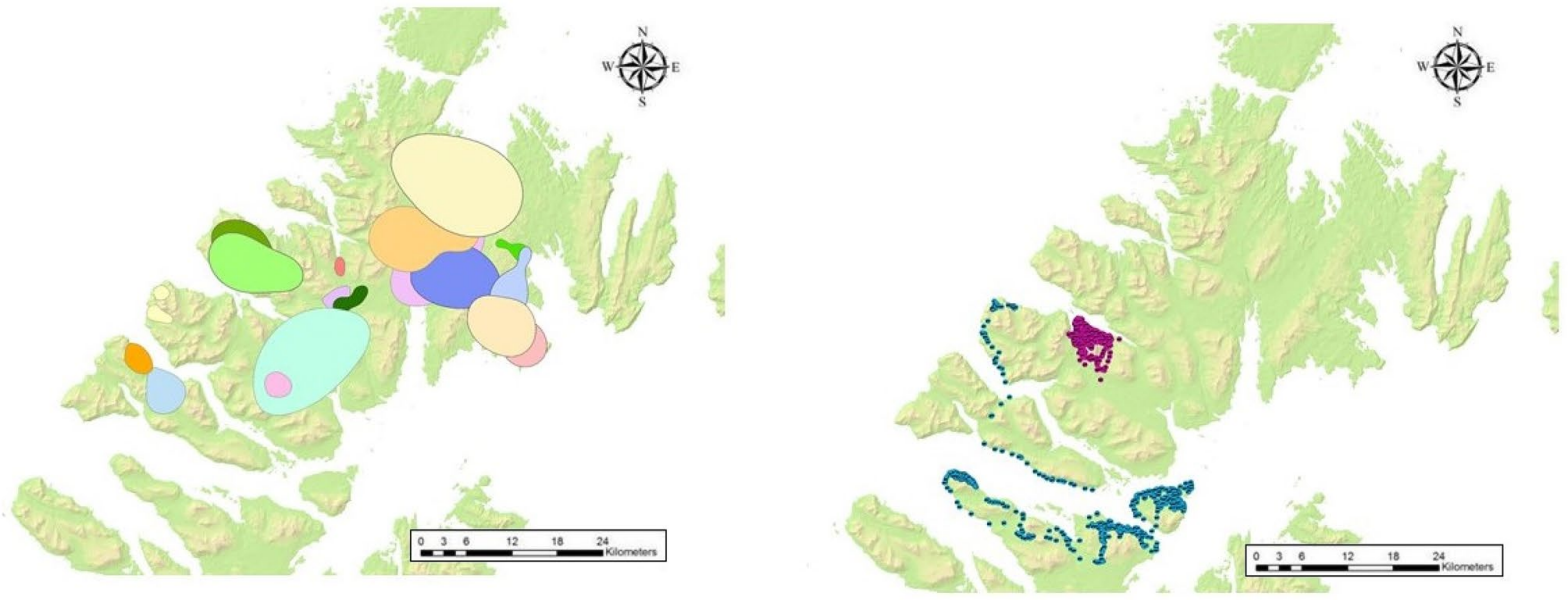
Fifty percent autocorrelated kernel density estimates for 18 GPS-collared brown bears during summer 2017 (left) and individual movements of two brown bears in summer 2020 (right), Afognak Island, Alaska, USA. Map generated with ArcGIS 10.5, ESRI 2020, https://www.arcgis.com/index.html.

We captured 141 bears on Afognak and Sitkalidak islands (44 male, 97 female) and obtained 333,727 locations for annual analysis, 63,428 spring locations, 184,439 summer locations, and 79,871 fall locations for seasonal analysis. Following subsampling, we conducted annual analysis for 77 individuals on Afognak Island (26 male, 51 female), and 42 individuals on Sitkalidak Island (8 male, 34 female) (Table 2). For seasonal analysis, we used data from 40 individuals in spring, 125 individuals in summer, and 78 individuals in fall (Fig. 3).

**Table 2:** Area (km^2^) of 50% utilization distributions of GPS-collared brown bears, Afognak and Sitkalidak islands, Alaska, USA, June–November 2017–2020.

Season	Island	Male				Female				Female with young			
		n	Median	Mean	SD	n	Median	Mean	SD	n	Median	Mean	SD
Annual	Afognak	26	22.2	39.3	43.0	22	6.8	38.4	73.8	29	8.5	18.5	22.5
Spring		12	24.0	37.6	38.4	8	4.2	5.0	3.0	10	5.6	11.1	15.9
Summer		27	21.7	46.2	52.4	18	5.2	35.9	70.4	36	9.7	20.9	31.6
Fall		10	66.8	74.8	58.6	14	3.7	8.7	10.1	15	4.7	12.3	17.0
Annual	Sitkalidak	8	39.1	68.2	64.5	20	7.9	8.8	8.0	14	5.0	46.8	80.9
Spring		3	112.9	117.2	81.4	6	4.2	4.9	2.7	1	8.6	8.6	–
Summer		7	19.1	51.6	51.7	21	9.1	10.0	9.9	16	4.9	35.7	64.3
Fall		5	36.4	53.0	48.7	20	3.8	9.2	12.1	14	5.4	37.6	80.7

Bear range sizes were not normally distributed with a median annual range size of 22.2 km^2^ for males and 6.8 km^2^ for females on Afognak, and 39.1 km^2^ for males and 7.9 km^2^ for females on Sitkalidak (Table 2). Afognak male annual ranges were overall 3.2 and 2.6 times larger than solitary females and females with young, respectively. Sitkalidak male annual ranges were on average 4.8 and 7.7 times larger than solitary females and females with young, respectively. Solitary females on Afognak Island used smaller UDs at all temporal scales compared to females with dependent young, whereas on Sitkalidak Island solitary females used larger UDs annually and during summer compared to females with dependent young.

From our RSFs, female elk selected for higher elevations in summer and exhibited selection for higher elevations during calving and lower elevations during hunting periods (Appendix, Table S1). Grassland, wetland, and dwarf shrub land covers were selected for during all seasons. Forest, non-vegetated and dwarf shrub habitats were often selected against, particularly during calving and summer. The model for hunting period 2019 was not included in further analysis due its low AUC score (0.57).

**Figure 4:**
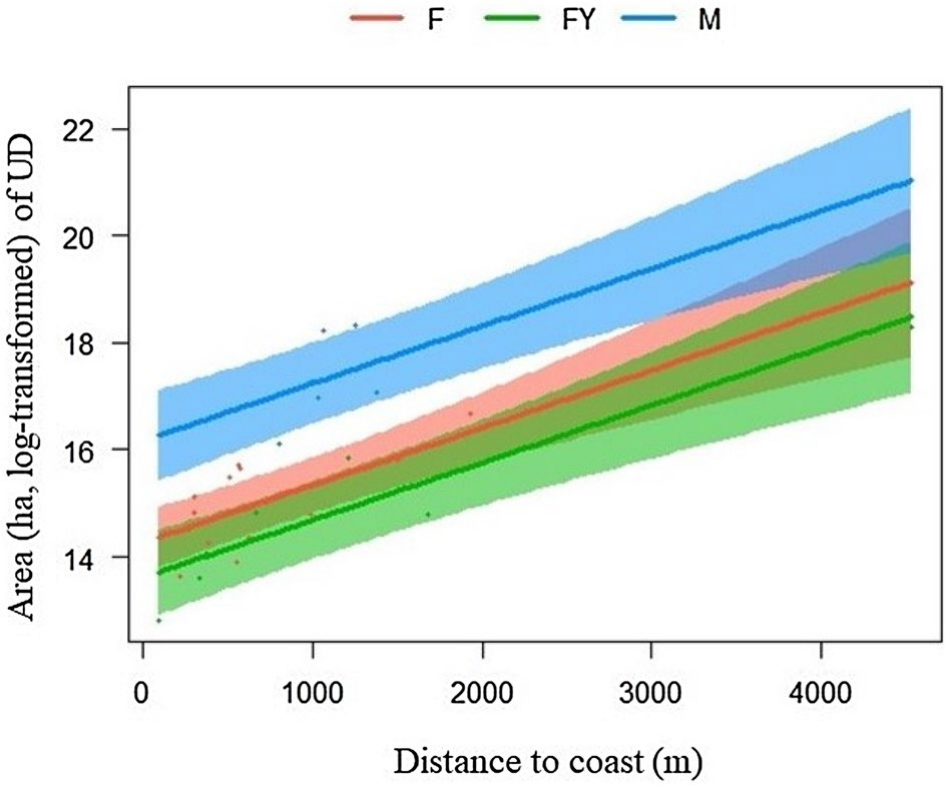
Relationship between log-transformed area of annual 50% utilization distributions, with 95% confidence intervals, and average distance to coast among reproductive classes of brown bears (solitary females [F], females with young [FY], males [M]), Sitkalidak Island, Alaska, USA, June–November 2017–2020.

Within our bear models we detected a strong correlation (> 0.70) between two pairs of covariates (1 = edge density and mean of patch area, 2 = total patch area and > 60 year old timber stands) and did not include both variables from the same pair within the same model. Top ranked annual models for Afognak supported the RDH (54% weight of evidence) while Sitkalidak supported the TRVH (85%) (Table 3). Distance from coast was most influential in the Sitkalidak model (p-value < 0.001, Table 4; Fig. 4), while timber stands 0–5 years old (p-value = 0.003) and 6–20 years old (p-value = 0.002) were most influential in the Afognak model.

**Table 3:** Selection results of 10 linear models using small-sample Akaike information criterion (AICc; Burnham and Anderson, 2002) to explain variation in area of annual and seasonal 50% utilization distributions (km^2^) of brown bears, Afognak and Sitkalidak islands, Alaska, USA, June–November 2017–2020. Refer to Table 1 for model parameter definitions.

Season	Island	Hypothesis	Model	AICc	W
Annual	Afognak	RDH	Timber stand ages + berry habitat + edge density + age + reproductive status + year	− 10.31	0.54
		RDH	Timber stand ages + berry habitat + mean of patch area + age + reproductive status + year	− 9.12	0.29
	Sitkalidak	TRVH	Distance to salmon stream + distance to coast + reproductive status + age + year	− 55.00	0.85
Spring	Afognak	Sex and age	Age + reproductive status	− 37.76	0.32
	Sitkalidak	TRVH	Distance to salmon stream + distance to coast + age + reproductive status + year	− 37.14	0.99
Summer	Afognak	RDH	Timber stand ages + berry habitat + edge density + age + reproductive status + year	− 19.52	0.37
			Timber stand ages + berry habitat + edge density + total patch area + age + reproductive status + year	− 18.32	0.20
	Sitkalidak	RDH	Shannon’s diversity + age + reproductive status + year	− 24.09	0.63
Fall	Afognak	TRVH	Probability of elk + reproductive status + age + year	− 16.84	0.81
	Sitkalidak	RDH	Berry habitat + total patch area + mean of patch area + age + reproductive status + year	− 9.04	0.37
			Berry habitat + edge density + total patch area + age + reproductive status + year	− 9.71	0.52

**Table 4:** Parameter estimates for annual and seasonal 50% utilization distribution (UD) variations for brown bears on Afognak and Sitkalidak islands, Alaska, USA, June–November 2017–2020. Refer to Table 1 for numbered model and parameter definitions. Numbers in bold represent significant values.

Season	Island	Top model	Variables	Estimate	Std. error	p-value
Annual	Afognak	RDH7	Intercept	7.49	39.30	0.849
			0–5	− 1.58	0.52	**0.003**
			6–20	1.70	0.52	**0.002**
			21–40	0.26	0.81	0.742
			41–60	0.62	0.37	0.101
			> 60	− 1.07	0.38	**0.007**
			Berry habitat	− 0.15	0.14	0.306
			Edge density	− 0.18	0.12	0.135
			Age	− 0.08	0.08	0.299
			Female	− 0.21	0.17	0.222
			Female with young	0.00	0.05	0.970
			Male	0.10	0.06	0.096
			Year	− 0.00	0.01	0.860
	Sitkalidak	TRVH1	Intercept	− 1.24	4.82	**0.012**
			Salmon stream	2.15	1.01	**0.038**
			Coast	8.05	1.20	**< 0.001**
			Age	7.27	8.29	0.384
			Female	− 6.75	4.16	0.871
			Female with young	− 4.34	4.17	0.304
			Male	2.43	4.77	**< 0.001**
			Year	6.19	2.38	**0.012**
Spring	Afognak	Sex and age	Intercept	0.50	0.03	**< 0.001**
			Age	0.03	0.08	0.667
			Female	− 0.09	0.04	**0.040**
			Female with young	− 0.07	0.04	0.093
			Male	0.15	0.03	**< 0.001**
	Sitkalidak	TRVH1	Intercept	− 170.93	41.18	**0.002**
			Salmon stream	0.29	0.10	**0.016**
			Coast	1.92	0.32	**< 0.001**
			Age	0.01	0.03	0.623
			Female	− 0.08	0.02	**0.003**
			Female with young	0.07	0.03	0.068
			Male	0.15	0.03	**0.002**
			Year	0.08	0.02	**0.001**
Summer	Afognak	RDH7	Intercept	22.57	38.18	0.556
			0–5	0.41	0.54	0.439
			6–20	− 0.51	0.53	0.336
			21–40	0.24	0.88	0.783
			41–60	0.29	0.34	0.387
			> 60	− 0.47	0.37	0.213
			Berry habitat	− 0.03	0.13	0.798
			Edge density	− 0.37	0.12	**0.002**
			age	0.05	0.08	0.536
			Female	− 0.08	0.04	**0.048**
			Female with young	− 0.00	0.03	0.822
			Male	0.06	0.04	0.126
			Year	− 0.01	0.01	0.568
	Sitkalidak	RDH5	Intercept	− 157.69	88.23	0.084
			Shannon’s diversity	0.38	0.11	**0.002**
			Age	0.06	0.11	0.579
			Female	− 0.02	0.04	0.527
			Female with young	0.02	0.06	0.646
			Male	0.10	0.06	0.128
			Year	0.07	0.04	0.083
Fall	Afognak	TRVH3	Intercept	102.14	49.26	**0.044**
			Elk RSF	− 0.35	0.09	**< 0.001**
			Female	− 0.13	0.04	**0.003**
			Female with young	0.01	0.04	0.806
			Male	0.22	0.04	**< 0.001**
			Age	− 0.06	0.09	0.447
			Year	− 0.05	0.02	**0.045**
	Sitkalidak	RDH7	Intercept	− 70.60	98.78	0.483
			Berry habitat	0.41	0.14	**0.009**
			Edge density	− 0.31	0.12	**0.016**
			Total patch area	0.56	0.14	**0.001**
			Age	0.14	0.11	0.222
			Female	− 0.03	0.04	0.445
			Female with young	− 0.12	0.06	0.062
			Male	0.21	0.09	**0.032**
			Year	0.03	0.04	0.480

Seasonal support for hypotheses varied. During spring, bears on Afognak did not demonstrate support for either hypothesis, instead the top model included age and reproductive status (Table 3). In contrast, the bear population on Sitkalidak during spring showed support for the TRVH with distance to coast most influential (p-value < 0.001, Table 4). Both populations showed support for the RDH in summer. On Afognak a decrease in habitat edge density lead to an increase in range size (p-value = 0.002, Table 4), while an increase in Shannon’s diversity index resulted in smaller range sizes on Sitkalidak (p-value = 0.002, Table 4). During fall, the Afognak population showed greater support for TRVH and the Sitkalidak population for RDH. Lower probability of elk occurrence on Afognak during fall resulted in larger bear UDs (p-value < 0.001, Table 4). On Sitkalidak, an increase in berry habitat and total patch area resulted in smaller UDs (p-value = 0.009 and 0.001, respectively, Table 4).

## Discussion

We found divergent patterns in brown bear annual and seasonal space use within two island populations, demonstrating marked ecological and behavioral plasticity, as documented previously on the Kodiak Archipelago^36^. Annual brown bear range sizes were influenced by the temporal availability of resources and their spatial distribution. Support for the TRVH occurred in the Sitkalidak population in spring, however contrary to our predictions both study populations supported the RDH in summer, likely due to an increase in range size to meet optimal nutritional gain. Both populations again contrasted in their support for either hypothesis in fall, and overall these results demonstrate divergences between optimal range sizes within and between temporal scales, likely dependent on available resources and landscape structure within each system.

At an annual scale the Sitkalidak brown bear population, inhabiting an island without forests or elk as potential prey, followed the TRVH. We found a positive correlation between brown bear annual range size and distance from coasts, where increasing distances from coasts resulted in larger ranges. This is likely attributed to bears traversing greater areas to meet nutritional demands when farther from coastal marine resources. Coastal intertidal habitats can provide important food for bears throughout the year, particularly following den emergence^51^. Marine invertebrates, clams, and kelp along with whale carcasses (which occur more frequently on Sitkalidak Island coastal shores than on Afognak Island) are potential important foods which may in part explain the increased importance of coastal areas for this island population^36,60^.

Contrary to our predictions, brown bear space use on Sitkalidak Island supported the TRVH in spring. Similar to annual space use, support for the TRVH on Sitkalidak during spring is likely attributed to the concentration of emerging vegetation and clumped coastal food resources^36,51,61^. Males from both islands occupied larger ranges than solitary females and females with young, likely attributed to mate-seeking behavior during the breeding season^62^. We also found support for the TRVH in the Afognak population in fall. Terrestrial meat is an important food for brown bears, particularly when vegetation and marine resources are limited, and is linked to increased body mass^63^. Brown bears prey on ungulate neonates to varying extents depending on species, availability, and location^64,65^. We did not find a relationship between the presence of female elk during calving (spring) and bear range size on Afognak, which may reflect population-level opportunistic foraging of elk calves, as observed with black bears (*Ursus americanus*) and neonatal ungulates^52,66,67^. However, during the elk hunting period (fall), a decrease in the probability of elk occurrence resulted in an increase in bear range size on Afognak Island. This is possibly due to bears scavenging on discarded elk carcasses^68^, and when less carrion is available bears increase their space use to obtain sufficient nutrients^69^. Alternatively, as brown bears are omnivorous, they may select for more vegetation-rich patches during this period, similar to areas selected for by elk^70^. We acknowledge the estimates from our elk RSF models contain no measured error and thus their inclusion within bear models warrant caution with any interpretation. However, such methods are frequently used in predator prey studies^71,72^. Support for TRVH at both annual and seasonal scales in these two populations appears in response to varied temporal concentrations of terrestrial meat, vegetation, and coastal food resources, suggesting the temporal availability of food resources is more influential on range size then their spatial distribution.

At an annual scale we found support for the RDH within the Afognak population, likely in part a result of greater spatial variability of resources due to increased habitat fragmentation from commercial timber harvest. Brown bears can select for or against areas with commercial timber harvest^48,73,74^. Clearcut areas can provide abundant berry-producing species, which can increase through 20 years following harvest^48,75^, but associated disturbance can disrupt denning^76^ and lead to increased hunting pressure^77^. We found that brown bear annual range sizes decreased with increased proportions of recent clearcuts (0–5 years old). An increase in proportions of older clearcut areas (6–20 years old) also resulted in larger range sizes. Similar selection of clearcuts by grizzly bears has been reported in other areas^73,78–81^ and suggests that clearcut areas on Afognak Island provided increased food for bears at this larger temporal scale. We also noted a positive relationship between proportions of older forests (> 60 years) and range sizes of bears on Afognak Island. Older-aged forests can provide abundant devil’s club that is consumed by brown bears, denning habitat^76^, and often occur adjacent to salmon streams on Afognak.

Contrary to our predictions, bears in both populations demonstrated support for the RDH in summer, a season of increased resource availability^36^. Following RDH, animals should increase their space use as resource patches become more dispersed throughout the environment^10^. Barnes^34^ also reported increased range sizes in summer among brown bears on southern Kodiak Island. Similar effects on space use were reported by Valeix et al.^82^, who noted that African lion (*Panthera leo*) movements followed the RDH, where pride range sizes increased as waterholes (a proxy for prey) became more dispersed. Although salmon and berries become more abundant throughout our study system during summer, their distributions are patchy. Optimal mass gain in brown bear occurs with mixed diets of meat (terrestrial and aquatic) and vegetation^83^, thus bears likely adhered to the RDH in summer as they moved between patches to gain more diverse nutritional resources^14^. Despite both populations supporting the RDH in summer, we noted divergent optimal behavioral strategies, where males on both islands adhered to an area minimization strategy, likely a result of their dominance over resource patches^20^. In comparison, solitary female space use supported a resource maximization strategy, while females with dependent young displayed variation between these two strategies depending on study location. This variation in strategy may reflect the number and age of offspring accompanying females, where females with younger offspring (cubs of the year) may avoid resource patches occupied by more dominant individuals^84^. Despite the importance of salmon in the diet and subsequent movement behavior of brown bears^27,33–35^, we did not find a relationship between the distance to salmon streams and bear range size. However, we suspect this is attributed to the large abundance and close spatial proximity of salmon streams throughout our study sites. In essence, bears were never far from a stream with salmon, reducing the importance of this covariate in our models. We also note that the increase in range sizes during summer may reflect increased movements among different salmon spawning areas, as bears respond to the temporal segregation of salmon runs in different watersheds^35^.

We found varied responses in brown bear range size to landscape heterogeneity in both populations during summer. Similar to Mangipane et al.^14^ who noted increased homogenous landscapes resulted in increased brown bear range sizes, we found that as patch edge density decreased (suggesting a more homogenous landscape), Afognak bear range sizes increased. Further supporting the ecological flexibility of this species, we found that Sitkalidak population range sizes were influenced by heterogeneous landscapes during the same period, where an increase in Shannon’s diversity index resulted in smaller range sizes. Smith and Pelton^85^ similarly noted black bear range size decreased with increased habitat diversity in Arkansas. However, increased landscape heterogeneity resulted in larger black bear range sizes in Missouri, USA^7^. We suspect the relationship between homogenous landscapes and increased range sizes in the Afognak population may be due to abundant food sources (e.g. berries, salmon) spatially dispersed among patches, increasing the need to traverse larger areas^36,86^. This relationship may also be the outcome of extensive timber harvest on Afognak, which has resulted in homogenous patches of young trees. These monoculture regeneration units likely do not consistently provide sufficient food for bears at these temporal scales^80^, resulting in larger range sizes. The variation in range size between homogenous and heterogeneous landscapes in both populations likely reflects changing abundances and distributions of important foods^36,81^.

The Sitkalidak population again supported the RDH in fall, where range sizes were influenced by habitat homogeneity, with decreasing edge density resulting in smaller ranges. This is potentially a result of the relationship with berry habitat found in this season, where an increase in this habitat type also resulted in reduced range sizes. Late salmon runs in this system are also likely an important resource monopolized by this population. Sitkalidak bears adhered to an area minimization strategy within the RDH and reduced their movements, perhaps to exploit habitats with both higher berry and salmon availability^81^. These results corroborate previous suggestions that animals reduce range sizes when there is high predictability of resource distribution across the landscape^1^. Support for the RDH in this population is likely the result of bears altering movements to exploit berry and salmon resources for optimal mass gain, similar to results found in the summer.

As predicted, we found males in both populations occupied larger ranges compared to females at both temporal scales. In polygynous mating systems, such as with brown bears, males often exploit larger areas to increase their reproductive opportunities during the breeding season^62^. Similar to studies on black bears^7,87^ and brown bears^21^, males in our study held larger ranges compared to solitary females and females with dependent young. However, bears on Afognak used larger median ranges in fall compared to spring (when mating occurs) whereas males on Sitkalidak maintained their largest median range during spring. Increased space use among males is likely a result of increased metabolic demands associated with larger body sizes in fall^21^ and mate-seeking behavior in spring^62^. Our sample size for male brown bears on Sitkalidak was lower than for males on Afognak, which may underestimate true range size variation in this population. We found that solitary females held smaller ranges than females with young on Afognak across multiple seasons. This result was opposite to what we predicted under an infanticide risk avoidance strategy^21^, and suggests that females with young have larger ranges to support their increased metabolic demands associated with cub rearing^19^. Although we found no differences in range size between solitary females and females with young on Sitkalidak Island, solitary females held larger average median ranges annually and in summer.

We found no evidence to support our predictions of age-based dominance resulting in larger ranges in brown bears. However, more dominant individuals may hold smaller and more productive ranges to monopolize resource rich areas^88^, particularly during salmon season for brown bears^20^. We suggest that future studies include age and body mass data to better examine whether larger and more dominant individuals exhibit divergent space use patterns. We acknowledge sampling limitations, particularly among the Sitkalidak population. We had lower sample sizes overall for males in this population, and low sample sizes for females with young during spring. However, despite these limitations and given our overall sample size and study duration, we suggest these patterns within an optimality framework are ecologically relevant.

Temporal and spatial heterogeneity are key ecological mechanisms structuring animal ranges^87^; we provide evidence in support of animals making behavioral choices to expand and contract ranges based on resource availability and demographic characteristics^88^. Few studies addressing home range estimation have fully considered range size dynamics, or the “how” and “why” of animal movement ecology^3^. We add to Nathan^3^ and Tao et al.^88^, who demonstrated optimality theory from a literature review on mammals and birds along with animal movement simulations, by demonstrating the benefits of combining optimality theory and utilization distributions to understand how internal and external states of animals influence range fluctuations. Our work builds on previous research conducted on the role of optimality theory in black bear space use^8,87^. Optimality theory provides a unique framework to examine periodic changes in range sizes in response to forage, and ultimately better understand the fitness motivated movement decisions made by animals^88^. The highly variable temporal support observed for RDH and TRVH within and between two island populations in a single metapopulation emphasizes the importance of scale and location considerations when examining animal spatial ecology. Throughout much of the world, human activities can markedly influence movements of animals through landscape alterations and hunting, as found in our study system. It is increasingly important to better understand spatio-temporal patterns and underlying mechanisms underpinning animal movement, coupled with anthropogenic influences, and their roles in ecological and evolutionary processes. In doing so, we can better design and implement management and conservation strategies which account for the variable responses of animals to changes within their environment.

## Supplementary Information

Supplementary Table S1.
