# Supplementary material for: Variable intraspecific space use supports optimality in an apex predator
Source: Sci Rep. 2021 Oct 26;11:21115. doi: 10.1038/s41598-021-00667-y (PMC8548348; doi:10.1038/s41598-021-00667-y)
Supplement: Supplementary file 1 — Supplementary Table S1. [file 41598_2021_667_MOESM1_ESM.pdf]

Variable intraspecific space use supports optimality in an apex predator

S. P. Finnegan[1], N. J. Svoboda[2], N. L. Fowler[1 3], S. L. Schooler[1], J. L. Belant[1]

1. Global Wildlife Conservation Center, State University of New York, College of Environmental Science and Forestry, Syracuse, NY, USA.

2. Alaska Department of Fish and Game, Kodiak, AK, USA.

3. Alaska Department of Fish and Game, Soldotna, AK, USA.

Corresponding author: S. P. Finnegan, 1 Forestry Drive, Syracuse NY, 13204, USA.

shannonfinnegan8@yahoo.com.

Supplementary material

Table S1. Parameter estimates from annual and seasonal resource selection functions for female collared elk (*Cervus canadensis roosevelti)*, Afognak Island, Alaska, USA, June–August 2017–2020.

**Season**	**Year**	**Variables**	**Estimate**	**Std. error**	**p-value**
Annual	2017	Intercept	-2.94	0.12	<2e-16
		Scale elevation	0.05	0.00	<2e-16
		Dwarf-shrub	1.42	0.11	<2e-16
		Forest	1.06	0.11	<2e-16
		Grassland	1.94	0.11	<2e-16
		Non-veg other	0.04	0.12	0.689
		Shrub	1.47	0.12	<2e-16
		Wetland	1.21	0.12	<2e-16
	2018	Intercept	-2.15	0.10	<2e-16
		Scale elevation	0.00	0.00	0.026
		Dwarf-shrub	0.87	0.10	<2e-16
		Forest	0.20	0.10	0.029
		Grassland	1.28	0.10	<2e-16
		Non-veg other	-0.61	0.10	4.20e-10
		Shrub	0.73	0.10	1.53e-14
		Wetland	0.81	0.10	<2e-16
	2019	Intercept	-1.85	0.13	<2e-16
		Scale elevation	-0.19	0.00	<2e-16
		Dwarf-shrub	0.72	0.13	9.03e-09
		Forest	-0.20	0.12	0.105
		Grassland	1.04	0.12	<2e-16

		Non-veg other	-1.03	0.13	3.60e-16
		Shrub	0.44	0.12	<0.001
		Wetland	0.62	0.13	9.47e-07
	2020	Intercept	-3.17	0.46	8.31e-12
		Scale elevation	0.01	0.01	0.623
		Dwarf-shrub	2.08	0.46	5.93e-06
		Forest	0.85	0.46	0.063
		Grassland	2.30	0.46	4.90e-07
		Non-veg other	0.22	0.46	0.635
		Shrub	1.77	0.46	<0.001
		Wetland	1.93	0.46	2.78e-05
Calving	2017	Intercept	-1.97	0.46	1.77e-05
		Scale elevation	0.48	0.04	< 2e-16
		Dwarf-shrub	0.48	0.42	0.256
		Forest	-0.63	0.42	0.136
		Grassland	1.30	0.42	<0.001
		Non-veg other	-1.37	0.46	0.003
		Shrub	0.54	0.42	0.197
		Wetland	1.19	0.45	0.008
	2018	Intercept	-3.12	0.47	5.13e-11
		Scale elevation	-0.50	0.01	< 2e-16
		Dwarf-shrub	1.77	0.48	<0.001
		Forest	0.93	0.47	0.051
		Grassland	2.46	0.47	2.11e-07
		Non-veg other	0.36	0.48	0.450
		Shrub	1.66	0.47	<0.001
		Wetland	1.97	0.48	3.44e-05
	2019	Intercept	-2.34	0.33	2.04e-12
		Scale elevation	-0.35	0.01	< 2e-16
		Dwarf-shrub	1.48	0.33	8.41e-06
		Forest	0.05	0.33	0.884
		Grassland	1.63	0.33	8.28e-07
		Non-veg other	-0.42	0.33	0.211
		Shrub	1.00	0.33	0.003
		Wetland	1.09	0.33	0.001
	2020	Intercept	-4.29	0.98	1.13e-05
		Scale elevation	0.32	0.02	< 2e-16
		Dwarf-shrub	2.60	0.97	0.007
		Forest	1.35	0.97	0.162
		Grassland	3.12	0.97	0.001
		Non-veg other	1.39	0.97	0.153
		Shrub	3.02	0.97	0.002
		Wetland	2.91	0.97	0.003
Summer	2017	Intercept	-2.77	0.15	< 2e-16
		Scale elevation	0.30	0.01	< 2e-16
		Dwarf-shrub	1.42	0.14	< 2e-16

		Forest	1.00	0.14	1.74e-13
		Grassland	1.67	0.14	< 2e-16
		Non-veg other	-0.07	0.14	0.637
		Shrub	1.14	0.14	< 2e-16
		Wetland	1.24	0.14	< 2e-16
	2018	Intercept	-1.80	0.16	< 2e-16
		Scale elevation	0.40	0.01	< 2e-16
		Dwarf-shrub	0.64	0.14	5.09e-06
		Forest	-0.10	0.14	0.48
		Grassland	0.73	0.14	2.32e-07
		Non-veg other	-1.37	0.15	< 2e-16
		Shrub	0.25	0.14	0.0754
		Wetland	0.46	0.15	0.0016
	2019	Intercept	-1.72	0.17	< 2e-16
		Scale elevation	0.24	0.01	< 2e-16
		Dwarf-shrub	0.56	0.16	0.00059
		Forest	-0.05	0.16	0.74877
		Grassland	0.67	0.16	3.05e-05
		Non-veg other	-1.67	0.17	< 2e-16
		Shrub	0.13	0.16	0.42235
		Wetland	0.46	0.17	0.00517
	2020	Intercept	-2.75	0.54	3.24e-07
		Scale elevation	0.28	0.02	< 2e-16
		Dwarf-shrub	1.86	0.53	0.000416
		Forest	0.27	0.53	0.612455
		Grassland	2.09	0.53	6.93e-05
		Non-veg other	0.25	0.53	0.63945
		Shrub	1.15	0.53	0.028884
		Wetland	2.23	0.53	2.91e-05
Hunting	2017	Intercept	-3.76	0.37	< 2e-16
		Scale elevation	-0.16	0.01	< 2e-16
		Dwarf-shrub	1.76	0.37	2.58e-06
		Forest	1.87	0.37	5.17e-07
		Grassland	2.73	0.37	2.13e-13
		Non-veg other	0.73	0.38	0.0523
		Shrub	2.38	0.37	1.68e-10
		Wetland	1.76	0.38	3.06e-06
	2018	Intercept	-3.32	0.30	< 2e-16
		Scale elevation	0.48	0.01	< 2e-16
		Dwarf-shrub	1.08	0.29	<0.001
		Forest	1.29	0.29	7.31e-06
		Grassland	2.30	0.29	1.16e-15
		Non-veg other	0.67	0.29	0.020432
		Shrub	1.95	0.29	9.97e-12
		Wetland	1.09	0.30	0.000213
